# Case Report: Leukoencephalopathy with calcifications and cysts caused by novel biallelic SNORD118 variants in a childhood-onset patient with long-term follow-up

**DOI:** 10.3389/fnins.2026.1865322

**Published:** 2026-10-07

**Authors:** Di Tian, Bin Chen, Wei Li, Junmei Wang, Zaiqiang Zhang

**Affiliations:** 1 Department of Neurology, Chuiyangliu Hospital Affiliated to Tsinghua University, Beijing, China; 2 Department of Neurology, Beijing Tiantan Hospital, Capital Medical University, Beijing, China; 3 Department of Pathology, Beijing Tiantan Hospital, Capital Medical University, Beijing, China

**Keywords:** cerebral microangiopathy, Labrune syndrome, leukoencephalopathy with calcifications and cysts, SNORD118, white matter disease

## Abstract

**Background:**

Labrune syndrome, also termed leukoencephalopathy with calcifications and cysts (LCC), is a rare autosomal recessive cerebral microangiopathy associated with pathogenic variants of the SNORD118 gene. Long-term clinical follow-up of childhood-onset LCC progressing into adulthood is limited in the literature.

**Case presentation:**

We present a 24-year-old woman whose neurological symptoms began at age 6 years with recurrent headaches and visual disturbances. Learning difficulties emerged at age 7 years. At age 22 years, she developed probable focal-onset seizures with bilateral tonic–clonic evolution, preceded by headache and blurred vision. At age 24 years, cognitive assessment showed a Mini-Mental State Examination score of 12, indicating significant impairment. Neuroimaging demonstrated the characteristic triad of diffuse white matter abnormalities, intracranial calcifications, and parenchymal cysts with fluid levels. Histopathological examination of a left frontal lobe biopsy demonstrated telangiectatic vessels, fibrinoid necrosis, hemosiderin deposition, and reactive gliosis, consistent with chronic cerebral microangiopathy. Whole-genome sequencing identified compound heterozygous SNORD118 variants, including the previously reported n.24C>T variant and the novel n.103G>C variant. Parental segregation confirmed that the two variants were inherited from different parents and were located in trans. The patient was treated with lacosamide 100 mg twice daily, with partial reduction in seizure frequency. Bevacizumab was discussed but declined for financial reasons.

**Conclusion:**

This case illustrates the long-term clinical course of childhood-onset LCC extending into adulthood and highlights the value of integrating characteristic neuroimaging, histopathology, and genetic testing. The patient’s longitudinal clinical history, together with pathological evidence of chro nic cerebral microangiopathy, is consistent with the progressive vascular phenotype previously described in LCC, although prospective radiological progression was not documented during the most recent follow-up. The novel SNORD118 n.103G>C variant was classified as Likely Pathogenic according to the applied ACMG/AMP framework; however, its pathogenic effect has not been experimentally validated.

## Introduction

Leukoencephalopathy with calcifications and cysts (LCC) is a rare neurological disease distinguished by three principal imaging features: widespread white-matter changes, intracranial calcifications, and brain parenchymal cysts. The condition was initially described in 1996 by Labrune et al. (1996) and is now recognized as a form of cerebral microangiopathy rather than a primary leukodystrophy. Subsequent clinicopathological studies have demonstrated vascular abnormalities, including telangiectasia, microhemorrhage, and fibrinoid necrosis (Livingston et al., 2014).

Later research demonstrated that LCC is caused by pathogenic mutations in SNORD118, a gene that encodes a small nucleolar RNA participating in ribosomal RNA maturation (Jenkinson et al., 2016). Although additional cases have been described in the literature, the clinical spectrum of LCC remains heterogeneous, and long-term observations of childhood-onset LCC progressing into adulthood are limited (Livingston et al., 2014).

What is unique about this case: To our knowledge, published reports of childhood-onset LCC with longitudinal clinical observation extending into adulthood remain limited. Our case is notable for an approximately 18-year interval from initial neurological symptoms to adult reassessment. Additionally, we report a novel SNORD118 variant (n.103G>C) that has not been previously described, thereby expanding the known mutational spectrum. The combination of long-term clinical follow-up, histopathological confirmation, and comprehensive ACMG-based variant classification provides a complete characterization of this rare disorder.

## Case presentation

### Patient information

A 24-year-old Chinese woman presented to our hospital with recurrent episodes of impaired consciousness preceded by headache and blurred vision. The patient had no significant past medical history other than the neurological symptoms described below. There was no consanguinity between her parents. Family history revealed that both parents were clinically healthy; her younger brother reported mild memory difficulties but remained functionally independent. He has not undergone genetic testing for SNORD118.

### Timeline of the episode of care

Table 1 presents the chronological timeline of the patient’s clinical course from symptom onset to the most recent follow-up.

**Table 1:** Clinical timeline.

Age	Event
6 years	First episode of transient headache with visual disturbance, lasting several minutes; cranial CT showed small intracranial calcifications
7 years	Learning difficulties emerged; persistently poor academic performance throughout primary school
15–21 years	Relatively stable period; no significant neurological deterioration reported
22 years	She experienced her first probable focal-onset seizure with bilateral tonic–clonic evolution while riding a bicycle, preceded by headache and blurred vision, with urinary incontinence and foaming at the mouth
22–24 years	Seizures recurred once or twice per month; progressive cognitive decline noted by family
24 years	Hospital admission for evaluation; MMSE score 12; neuroimaging and biopsy performed; genetic testing completed; lacosamide initiated

### Clinical findings

On admission, neurological examination revealed normal muscle strength, sensation, and gait. Cognitive assessment using the Mini-Mental State Examination yielded a score of 12, indicating significant cognitive impairment. Ophthalmological examination was unremarkable. Electroencephalography showed sharp and slow waves in the bilateral occipital and parietal regions.

### Diagnostic assessment

Cranial CT demonstrated widespread intracranial calcifications involving the bilateral basal ganglia (Figure 1A) and cerebellum (Figure 1B), as well as two cystic lesions with fluid levels. Brain MRI demonstrated two large cystic lesions located in the right thalamus and parietal lobe on T1-weighted images (Figure 1C) and diffuse hyperintensities in the periventricular and deep white matter on T2-weighted (Figure 1D) and FLAIR sequences (Figure 1E). No diffusion restriction (Figure 1F) or contrast enhancement was observed (Figure 1G).

**Figure 1:**
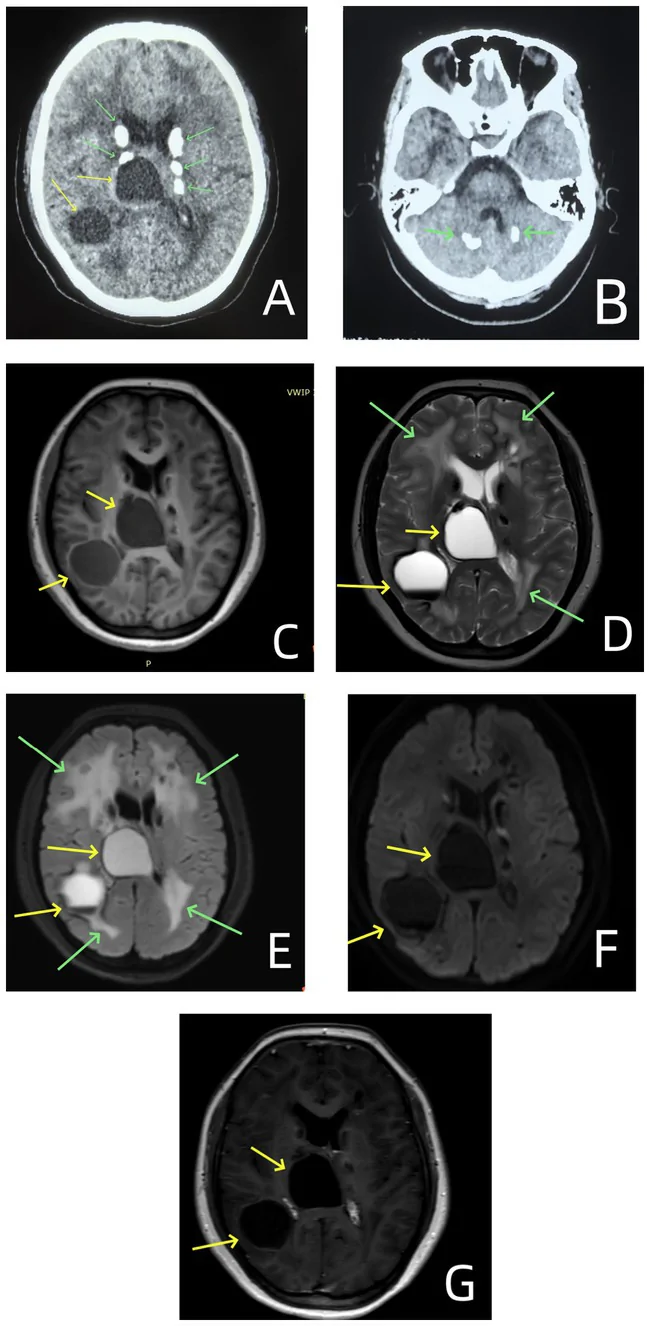
Neuroimaging findings. Non-contrast cranial computed tomography (CT) revealed multiple asymmetric intracranial calcifications involving the bilateral basal ganglia **(A)** and cerebellum **(B)**. In addition, two cystic lesions with fluid levels were observed (yellow arrows). Brain magnetic resonance imaging (MRI) demonstrated two large cystic lesions located in the right thalamus and parietal lobe on T1-weighted images **(C)**. T2-weighted **(D)** and fluid-attenuated inversion recovery (FLAIR) images **(E)** showed diffuse hyperintensities in the bilateral periventricular and deep white matter (green arrows). Notably, fluid levels were present within both cysts. Diffusion-weighted imaging showed no restricted diffusion within the cystic lesions **(F)**. Contrast-enhanced T1-weighted images revealed no enhancement of the cyst walls or surrounding white matter **(G)**.

A CT-guided needle biopsy of the left frontal lobe was performed. Histopathological examination demonstrated telangiectatic dilatation of small vessels, fibrinoid necrosis, hemosiderin deposition (Figures 2A,B), scattered calcifications (Figure 2C), angiomatoid vascular proliferation (Figure 2D), consistent with chronic vascular pathology.

**Figure 2:**
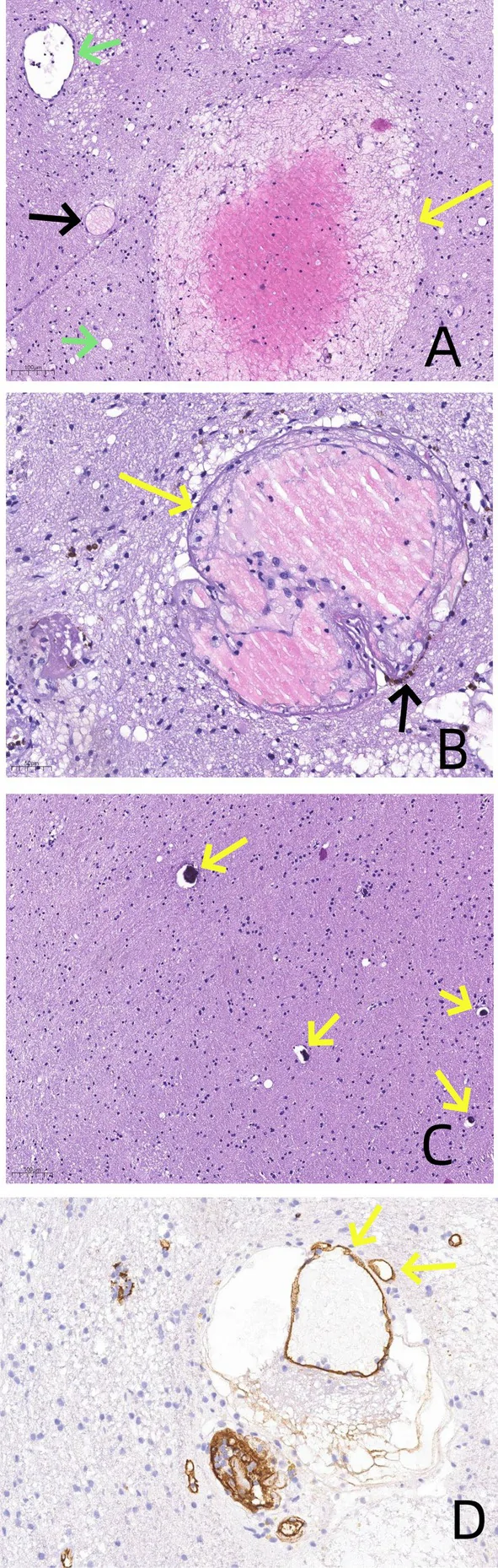
Histopathological findings. Hematoxylin and eosin staining demonstrated focal hemorrhage (yellow arrow), telangiectatic dilatation of small vessels (black arrow), and multiple cystic cavity formations (green arrow) **(A)** (×10). At higher magnification, angiomatoid vascular proliferation was evident (yellow arrow), accompanied by hemosiderin deposition (black arrow) **(B)** (×20). Scattered calcium deposits were also observed within the parenchyma **(C)** (×10). Immunohistochemical staining showed prominent vascular proliferation highlighted by CD34 **(D)** (×20).

Genetic analysis identified compound heterozygous variants (chr17:8076882, n.24C>T and chr17:8076803, n.103G>C) in the SNORD118 gene, each inherited from one parent. The n.24C>T variant has been previously reported in patients with LCC, supporting its established association with the disease (Wang et al., 2022; Iwama et al., 2017; Sim et al., 2022). While the variant n.103G>C has not been previously reported in the literature or documented in publicly available databases. ACMG/AMP classification designated n.24C>T as Pathogenic and n.103G>C as Likely Pathogenic (see Discussion for full classification details).

### Therapeutic intervention

The patient was treated with lacosamide at a dosage of 100 mg twice daily for seizure control. Given the focal-onset semiology with bilateral tonic–clonic evolution and the absence of persistent headache, mass effect-related symptoms, or focal neurological deficits attributable to the cystic lesions, neurosurgical intervention was not recommended.

Bevacizumab treatment was discussed with the patient and her family because of its potential therapeutic role in LCC; however, the family declined treatment for financial reasons. Therefore, the efficacy of bevacizumab could not be evaluated in this patient.

### Follow-up and outcomes

At three-month follow-up after lacosamide initiation, the patient reported a reduction in seizure frequency from 1 to 2 per month to one seizure every 2 months. No adverse effects of lacosamide were reported. Cognitive function remained stable but did not improve.

## Discussion

The present case illustrates the long-term clinical course of childhood-onset LCC extending into adulthood, characterized by early learning difficulties followed by the later emergence of cognitive impairment and epilepsy. The combination of characteristic neuroimaging and histopathological evidence of telangiectatic vascular abnormalities, fibrinoid necrosis, and hemosiderin deposition is consistent with the chronic cerebral microangiopathy described in LCC (Labrune et al., 1996; Livingston et al., 2014). The interpretation of the patient’s cognitive trajectory requires particular caution. Learning difficulties were already evident at approximately 7 years of age, with persistently poor academic performance during primary school, suggesting that cognitive dysfunction was present during childhood. At 24 years of age, her MMSE score was 12, with prominent impairment in memory, calculation, and visuospatial abilities, indicating severe global cognitive dysfunction. However, MMSE alone cannot reliably distinguish developmental cognitive impairment from subsequent acquired cognitive decline, particularly in a patient with longstanding learning difficulties and a potential developmental floor effect.

Importantly, family members reported further deterioration in memory and daily cognitive function during early adulthood, particularly after the onset of recurrent seizures. This history raises the possibility of superimposed acquired cognitive decline on a background of longstanding developmental or disease-related cognitive impairment. Nevertheless, because formal neuropsychological testing was not performed and serial standardized cognitive assessments were unavailable, the timing, magnitude, and relative contribution of developmental versus acquired cognitive impairment cannot be determined with certainty. Therefore, the term “progressive cognitive decline” should be interpreted cautiously in this case. Longitudinal assessment using standardized neuropsychological batteries would be required to more precisely characterize the cognitive trajectory in childhood-onset LCC.

### Treatment considerations

There is currently no established disease-modifying therapy for LCC, and management remains largely individualized according to the dominant clinical manifestations. Antiseizure medications are used for seizure control, whereas neurosurgical intervention may be considered in selected patients with symptomatic cysts, mass effect, or focal neurological deterioration (Novegno et al., 2024). In patients with clinically significant cystic lesions or progressive disease, bevacizumab has emerged as a potential therapeutic option. Several reports have described radiological and/or clinical improvement following treatment, including reduction or stabilization of cystic lesions and improvement in associated neurological manifestations (Fay et al., 2017; Martínez-Matilla et al., 2020; Scaffei et al., 2023). Nevertheless, the current evidence is based predominantly on individual case reports and small longitudinal series, and treatment response appears to be heterogeneous. Therefore, the potential benefits of bevacizumab should be balanced against its adverse effects, treatment burden, cost, and the uncertainty regarding long-term efficacy.

In our patient, bevacizumab was discussed but declined because of financial constraints. Consequently, no conclusions regarding its efficacy can be drawn from this case. The relatively short follow-up after lacosamide initiation also limits interpretation of seizure-control efficacy. Longer-term clinical and radiological follow-up will be important to determine whether symptomatic improvement is sustained and whether disease progression can be modified.

### Genetic analysis and ACMG classification

Genetically, we identified compound heterozygous variants in SNORD118, consisting of n.24C>T and n.103G>C. The n.24C>T variant has been previously identified in individuals with LCC and has been reported in both case-based and broader genetic studies of SNORD118-related disease (Wang et al., 2022; Iwama et al., 2017; Sim et al., 2022). The recurrence of this variant in affected individuals, together with its occurrence in trans with another disease-associated allele in the present case, provides additional support for its pathogenicity. In contrast, the n.103G>C variant has not been previously reported in the literature or documented in publicly available databases, thereby expanding the known mutational spectrum of LCC.

We applied the ACMG/AMP 2015 framework (Richards et al., 2015), while interpreting the computational and phenotype-based evidence in the context of SNORD118, a non-protein-coding small nucleolar RNA.

ACMG classification for n.24C>T (NR_033294.2(SNORD118):n.24C>T):

PM2: Absent from controls in population databases – Yes.

PM3: Detected in trans with a pathogenic variant – Yes.

PP3: Computational evidence supports deleterious RNA structure effect – Yes.

PP4: Phenotype highly specific for LCC – Yes.

PS4_Supporting: Previously reported in affected individuals with LCC (Wang et al., 2022; Iwama et al., 2017; Sim et al., 2022) – Yes.

→ Classification: Pathogenic

ACMG classification for n.103G > C (NR_033294.2(SNORD118):n.103G > C):

PM2: Absent from controls – Yes.

PM3: Detected in trans with a pathogenic variant – Yes.

PP3: Computational evidence supports deleterious RNA structure effect – Yes.

PP4: Phenotype highly specific for LCC – Yes.

→ Classification: Likely Pathogenic

### Comparison with similar and contrasting cases

Previous reports have described variable clinical courses in LCC. Some patients exhibit rapid neurological deterioration, while others remain stable for decades (Livingston et al., 2014; Jenkinson et al., 2016). Our patient’s course—childhood onset with mild early symptoms followed by progressive cognitive decline and epilepsy in early adulthood—represents an intermediate phenotype. Compared with the late-onset case reported by Wang et al. (2022), in which symptoms began at age 53 years, our patient had a much earlier presentation, suggesting that genotype–phenotype correlations in SNORD118-related LCC remain poorly defined.

### Strengths and limitations

The major strengths of this report include the approximately 18-year longitudinal clinical history extending from childhood to adulthood, multimodal neuroimaging, histopathological evidence of chronic cerebral microangiopathy, and identification of a previously unreported SNORD118 variant.

Limitations include: (1) limited post-treatment follow-up data for lacosamide, precluding assessment of long-term efficacy; (2) bevacizumab was not administered, so we cannot evaluate its potential benefit in this patient; (3) functional studies to confirm the pathogenic effect of the novel n.103G>C variant on U8 snoRNA expression or rRNA processing were not performed; (4) neuropsychological testing beyond the MMSE was not completed.

### Take-home lessons

From this case, we highlight three key lessons for clinicians: (1) LCC should be considered in patients with childhood-onset intracranial calcifications and white matter abnormalities, even when symptoms remain stable for years; (2) long-term follow-up is essential as cognitive decline and epilepsy may emerge in adulthood; (3) When the characteristic radiological triad of LCC is present, molecular genetic testing for SNORD118 should be prioritized and may obviate the need for invasive brain biopsy in diagnostically typical cases. Brain biopsy should generally be reserved for cases in which the diagnosis remains uncertain or an alternative diagnosis requiring histopathological confirmation cannot be adequately excluded.

### Patient perspective

The patient reported that her progressive memory difficulties and recurrent seizures have significantly impacted her daily living and employment opportunities. She expressed gratitude for the definitive genetic diagnosis, which helped her family understand the nature of her condition. She stated: “For many years, my family did not know why I was having these problems. Now that we have a name for the disease, it gives us some peace even though there is no cure.” She declined bevacizumab treatment primarily due to financial concerns, as the medication is not covered by medical insurance in her region. She hopes that reporting her case will raise awareness of this rare disease and facilitate access to targeted therapies for other affected individuals.

## Conclusion

This case expands the known mutational spectrum of SNORD118-associated LCC and illustrates the long-term clinical course of childhood-onset disease into adulthood. The combination of characteristic neuroimaging and histopathological findings is consistent with the cerebral microangiopathy underlying LCC. The ACMG-based classification provides a standardized framework for variant interpretation, with n.24C>T classified as Pathogenic and n.103G>C as Likely Pathogenic.

## Data Availability

The whole-genome sequencing data analyzed in this case were generated by an external research institution as a complimentary testing service. The authors received only a printed genetic testing report and did not obtain the original raw sequencing data or complete electronic genomic files. Therefore, the underlying sequencing dataset is not available for public deposition. The relevant genetic findings are reported in the manuscript. Supporting documentation from the original genetic testing report may be available from the corresponding author upon reasonable request, subject to patient confidentiality and institutional requirements.
